# Innovative Strategies for Upcycling Agricultural Residues and Their Various Pharmaceutical Applications

**DOI:** 10.3390/plants13152133

**Published:** 2024-08-01

**Authors:** Ludovic Everard Bejenaru, Antonia Radu, Adina-Elena Segneanu, Andrei Biţă, Costel-Valentin Manda, George Dan Mogoşanu, Cornelia Bejenaru

**Affiliations:** 1 Department of Pharmacognosy & Phytotherapy, Faculty of Pharmacy, University of Medicine and Pharmacy of Craiova, 2 Petru Rareş Street, 200349 Craiova, Romania; ludovic.bejenaru@umfcv.ro (L.E.B.); andreibita@gmail.com (A.B.); george.mogosanu@umfcv.ro (G.D.M.); 2 Department of Pharmaceutical Botany, Faculty of Pharmacy, University of Medicine and Pharmacy of Craiova, 2 Petru Rareş Street, 200349 Craiova, Romania; antonia.radu@umfcv.ro (A.R.); cornelia.bejenaru@umfcv.ro (C.B.); 3 Institute for Advanced Environmental Research, West University of Timişoara (ICAM–WUT), 4 Oituz Street, 300086 Timişoara, Romania; 4 Department of Analytical and Instrumental Chemistry, Faculty of Pharmacy, University of Medicine and Pharmacy of Craiova, 2 Petru Rareş Street, 200349 Craiova, Romania; valentin.manda@umfcv.ro

**Keywords:** agricultural residues, upcycling, sustainable waste management, environmental impact, innovative strategies, pharmaceutical applications

## Abstract

This review investigates innovative strategies for upcycling agricultural residues into valuable pharmaceutical compounds. The improper disposal of agricultural residues contributes to significant environmental issues, including increased greenhouse gas emissions and ecosystem degradation. Upcycling offers a sustainable solution, transforming these residues into high-value bioproducts (antioxidants, antitumor agents, antidiabetic compounds, anti-inflammatory agents, and antiviral drugs). Nanotechnology and microbial biotechnology have a crucial role in enhancing bioavailability and targeted delivery of bioactive compounds. Advanced techniques like enzymatic hydrolysis, green solvents, microwave processing, pyrolysis, ultrasonic processing, acid and alkaline hydrolysis, ozonolysis, and organosolv processes are explored for their effectiveness in breaking down agricultural waste and extracting valuable compounds. Despite the promising potential, challenges such as variability in residue composition, scalability, and high costs persist. The review emphasizes the need for future research on cost-effective extraction techniques and robust regulatory frameworks to ensure the safety, efficacy, and quality of bioproducts. The upcycling of agricultural residues represents a viable path towards sustainable waste management and production of pharmaceutical compounds, contributing to environmental conservation and public health improvements. This review provides an analysis of the current literature and identifies knowledge gaps, offering recommendations for future studies to optimize the use of agricultural residues in the drug industry.

## 1. Introduction

The improper disposal of agricultural residues via negligent practices is a significant environmental concern and negatively impacts ecosystems. Untreated and underutilized agricultural residues generate numerous greenhouse gas emissions, leading, through various mechanisms, to intensified climate change and the release of detrimental gaseous byproducts. Upcycling, the process of transforming waste materials into higher-value products, offers a sustainable solution by converting these residues into valuable bioproducts, including pharmaceuticals, such as antioxidants, antitumor agents, antidiabetic compounds, anti-inflammatory agents, and antiviral drugs [1].

The agricultural sector is a significant contributor to greenhouse gases, such as carbon dioxide, nitrous oxide, and methane, and the accelerating global production of residues inflicts a powerful effect on the environment, endangering human health and ecosystem longevity. These recent circumstances make urgent sustainability changes in current agricultural procedures and residue management policies mandatory [2,3]. From a pharmaceutical perspective, nanotechnology and microbial biotechnology are essential in transforming agricultural residues into enzymes and bioactive compounds, with improved bioavailability and targeted delivery to specific physiological sites, extending their applications in drug development [4,5]. Nanoencapsulation of bioactive substances in various states, employing matrices or inert nanostructured materials with unique properties, facilitates advanced drug delivery systems, enabling precise control over their therapeutic effects [6,7].

This study is important as it investigates innovative strategies for upcycling agricultural residues into valuable pharmaceutical compounds, addressing both environmental and economic challenges by transforming waste into high-value products. It highlights the benefits to the pharmaceutical industry through a renewable source of bioactive compounds, aiding in environmental conservation and public health. This review defines and classifies agricultural residues, details their unique properties, and identifies knowledge gaps such as variability in composition and scalability of biotechnological processes. It also recommends future research on cost-effective extraction techniques and regulatory frameworks, providing an in-depth critical analysis of the current literature on the effectiveness and scalability of various upcycling strategies. Previous studies have extensively documented the potential of agricultural residues as sources of bioactive compounds, focusing on their composition, potential applications, and extraction technologies like enzymatic hydrolysis, green solvents, microwave processing, pyrolysis, ultrasonic processing, acid and alkaline hydrolysis, ozonolysis, organosolv processes, and fermentation. These technologies enhance the breakdown of agricultural waste, facilitating the extraction of valuable compounds and promoting environmentally friendly utilization. However, a comprehensive analysis addressing challenges such as variability in residue composition and scalability is needed. Despite the promising applications and advancements in nanotechnology and microbial biotechnology, most research remains confined to laboratory settings, highlighting the necessity for targeted research and policy development to address high costs, regulatory challenges, and environmental impacts.

## 2. Characterization of Agricultural Residues

Agricultural residues, often regarded as waste, include byproducts generated from the harvesting and processing of crops and livestock, such as crop stalks, fruit peels, animal manure, and food processing waste, with increased concentrations of chemical substances [8,9].

These residues can be categorized into several types based on their origin and characteristics, the major ones being delineated in Table 1 [2,10].

Across the food supply chain, considerable residues result from diverse sectors, including the beverage industry, dairy and ice cream production, and fruit and vegetable processing [11].

Agriculture produces a daily average of 23.7 million food tons worldwide [12]. In the EU and globally, substantial volumes of agricultural and food processing residues are generated. Europe produces around 4,000,000 tons of tomato pomace annually, while the USA generates enormous amounts of orange peel waste. Other notable examples include 50,000–100,000 tons of vegetable oil waste in the UK, 57,000 tons of wheat straw in the USA, and 2,881,500 tons of olive pomace worldwide, highlighting the extensive global impact of these residues [10,13].

Table 2 provides an overview of various waste categories generated from food processing and agricultural activities accompanying specific examples [10].

Because of their valuable content of bioactive compounds, such as polyphenols, proteins, and carbohydrates, agricultural residues display a great potential for the pharmaceutical industry, as a source material for novel products, and, therefore, they are not considered a waste. Bioproducts are obtained through efficient and cost-effective pretreatment technologies (chemical, physical, and biological) and conversion procedures designed to improve the biochemical characteristics of the agro-residue biomass [14,15].

Lignin, cellulose, and hemicellulose are the three main organic components of agricultural biomass. Depending on their origin, crops and agro-industrial residues are the most substantial categories, being generated in enormous amounts each day, followed by aquaculture waste. As a result of their increasing volumes, there is a recent demand for creating optimal management strategies [16,17,18,19,20,21].

Different analytical characterization methods of agricultural residues are used by researchers, such as spectroscopic techniques [nuclear magnetic resonance (NMR), Fourier-transform infrared spectroscopy (FTIR)], chromatographic techniques [gas chromatography–mass spectrometry (GC–MS), high-performance liquid chromatography (HPLC)], and thermal analysis [thermogravimetric analysis (TGA), differential scanning calorimetry (DSC)]. For example, Rambo et al. investigated agricultural biomass from Brazil and explored the physical and chemical properties using X-ray diffraction (XRD), proximate and ultimate analyses, TGA, calorific value determination, near-infrared (NIR) spectroscopy, ultraviolet (UV) spectroscopy, high-performance anion exchange chromatography–pulsed amperometric detection (HPAEC–PAD), and accelerated solvent extraction (ASE) [22].

To summarize, agricultural residues have unique physicochemical properties; therefore, they are valuable for biofuel production due to their lignocellulosic composition, beneficial for composting and soil amendment because of their high nutrient content, and have pharmaceutical potential due to their bioactive compounds like polyphenols, flavonoids, and other antioxidants.

## 3. Upcycling Strategies for Agricultural Residues

Innovative pretreatment and conversion processes are imperative for successful agricultural residue conversion into useful biocompounds. Essential techniques such as enzymatic hydrolysis, green solvents, pretreatment, and advanced chemical treatments are used to break down complex biomolecules of lignocellulosic materials into simpler forms that can be utilized in drug development and other pharmaceutical applications [23,24,25].

Table 3 describes several examples of the latest upcycling strategies used to transform agricultural residues into valuable bioproducts with specific applications in the pharmaceutical sector, highlighting how these techniques contribute to the extraction and preparation of bioactive compounds for medicinal purposes.

### 3.1. Relevance for Bioeconomy

**Figure 1:**
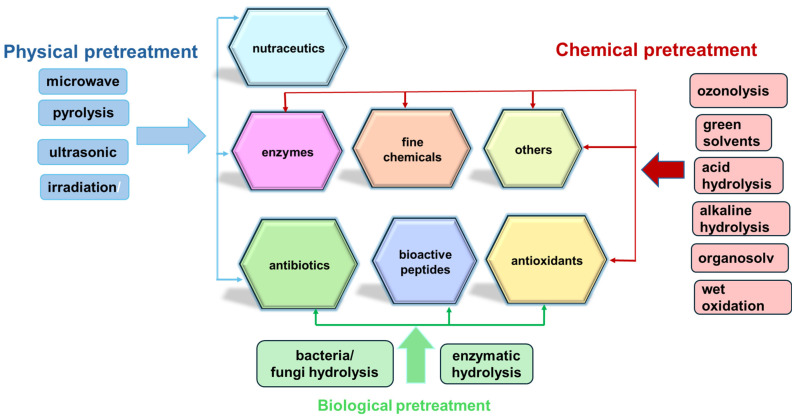
Schematic representation of valuable products obtained from agriculture residues.

The valorization of agricultural residues into upcycled products, such as bioactive compounds, antioxidants, nutraceuticals, and fine chemicals, represents a sustainable and economically viable alternative to conventional waste management methods like landfilling and incineration [44]. Furthermore, utilizing agro-waste as a substrate in various biotechnological and chemical processes allows for the recovery of a wide range of compounds crucial to the pharmaceutical industry (Figure 1), including antibiotics, industrial enzymes, and bioactive peptides [45].

These processes, integrating physical, chemical, and biochemical stages that prevent microbiological hazards, are designed to extract and enhance the utility of bioactive molecules, which are instrumental in drug development and other pharmaceutical applications [11]. Table 3 provides some examples of the effective valorization methods of agricultural residues for valuable bioactive compounds extraction, encompassing cascade or on-site processing of seasonal leftovers, industrial symbiosis, and conversion via green chemical or biotechnological methods. These approaches contribute to diminishing the dependence on fossil resources and support industrial development through sustainable resource management, highlighting the crucial role of integrated biorefining and the exploitation of renewable carbon sources in advancing pharmaceutical, cosmetic, and nutraceutical innovations [46].

### 3.2. Biorefinery Concept

The efficient upcycling of byproducts generated during biomass production holds significant potential for the pharmaceutical industry. The biorefinery concept, immediately endorsed by the scientific community as a sustainable alternative, involves recycling abundant and inexpensive agricultural wastes to produce commercially useful biomaterials, such as secondary chemicals, single-cell protein (microbial biomass), organic acids, enzymes, and biopolymers through advanced biotechnological techniques, like composting, fermentation, and anaerobic digestion [47,48]. Utilizing residues in chemical synthesis can improve public awareness, promoting a circular economy, with limitless supplies. From a pharmaceutical perspective, the biorefinery notion offers promising environmental benefits, including reduced greenhouse gas emissions, decreased disposable quantity of waste, and diminished reliance on fossil-based sources for raw material production [49,50].

Enzymes, essential components in numerous pharmaceutical processes and products, possess substantial industrial relevance due to their substrate specificity, ability to operate under medium reaction circumstances, minimal byproduct generation, and increased yield efficiency. However, the cost of raw materials accounts for up to a third of the total production expense for enzymes. Consequently, agricultural residue utilization presents a viable strategy for decreasing the costs, amounts of waste, and unfavorable environmental repercussions of their removal [51]. Numerous studies investigate the retrieval of enzymes from agro-residues. For instance, solid-state fermentation using *Bacillus coagulans* has been employed for lipase from melon waste recovery. Additionally, glucoamylase has been extracted through submerged fermentation with *Aspergillus niger*, and α-amylase has been obtained from coffee wastes via solid-state fermentation with the fungal strain *Neurospora crassa* [52,53,54].

The production of organic acids (such as butyric, lactic, acetic, and citric acids) through acidogenesis depends on the agricultural residue content [55]. Simultaneous saccharification and fermentation over two days produced significant quantities of organic acids from cabbage waste, applying Lactobacillus [56].

From a pharmaceutical perspective, single-cell protein derived from microbial fermentation of agricultural residues presents a valuable alternative protein source. Emerging innovative protein solutions are required due to the rising concerns about overpopulation growth and malnutrition. Single-cell protein, extracted from microbial biomass such as fungi, algae, or bacteria, provides a cost-effective alternative to regular protein sources. This can address protein deficiency and improve nutritional outcomes in medical nutrition therapy and dietary supplements [57,58].

Biopolymers from agricultural residues are significant due to their key properties, such as biocompatibility, biostability, biodegradability, and biofunctionality, and a wide range of applications in various industries, including pharmaceuticals, medicine, and cosmetics. An environmentally sustainable solution that not only contributes to reducing plastic waste but also agro-waste, is the production of bioplastics, such as polyhydroxyalkanoate (PHA) and polyhydroxybutyrate (PHB), which are organic polymers that degrade completely into carbon dioxide and water within months [59,60,61].

### 3.3. Extraction Methods

**Figure 2:**
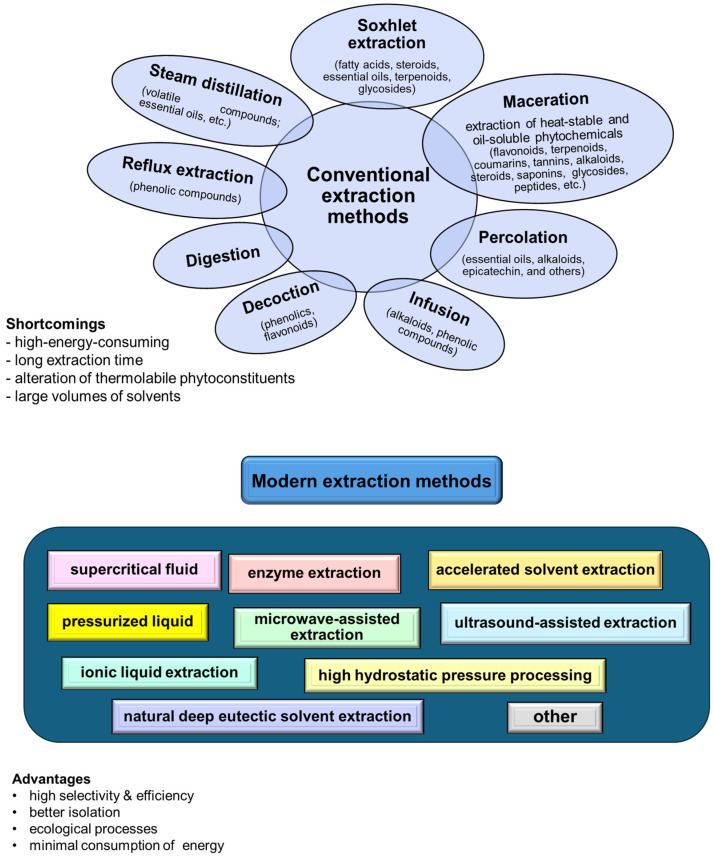
Schematic representation of the main extraction methods.

The extraction techniques for valuable compounds from agricultural residues involve both conventional and novel designs (Figure 2).

Traditional methods, such as hydrodistillation, maceration, and solvent or Soxhlet extraction, use organic solvents and require temperature and agitation conditions [62]. Furthermore, they generally require long extraction times and large solvent volumes, and may result in significant quantities of toxic waste [63].

Nonconventional or modern techniques (including supercritical fluid, pressurized liquid, and enzyme/ultrasound/microwave-assisted extractions) offer more efficient and environmentally friendly alternatives. These methods reduce solvent consumption and extraction time while increasing extraction efficiency and are better suited for preserving the integrity of bioactive compounds [64,65]. The advanced extraction methods are particularly beneficial as they allow for the recovery of valuable biobased molecules, natural biopolymers, and phytochemicals from agricultural residues, which can be used in drugs, cosmetics, nutraceuticals, and other therapeutics formulation. Additionally, eco-friendly solvents, such as deep eutectic solvents and natural deep eutectic solvents, enhance the green guarantees of the extraction processes. These solvents are highly effective in dissolving biomasses and have been successfully applied to extract valuable components from various agricultural residue sources, offering a sustainable approach to waste valorization in the pharmaceutical industry [66,67,68].

## 4. Innovative Techniques for Recovery of Bioactive Compounds from Agricultural Residues

The substances derived from agro-waste hold remarkable potential for pharmaceutical applications due to their diversified and important properties. These compounds, including polyphenols, vitamins, minerals, fatty acids, and antioxidants, can be isolated from agricultural residues such as fruit peels, vegetable wastes, and other byproducts. They exhibit various pharmacological activities, including antioxidant, anti-inflammatory, antibacterial, and antitumor effects, making them beneficial for developing nutraceuticals, nutritional supplements, and drugs. These biochemicals can enhance health benefits, improve dietary profiles, and provide sustainable alternatives to synthetic compounds in pharmaceutical products. For instance, antioxidants extracted from fruit peels can be used to develop anti-inflammatory and anticancer drugs. At the same time, phenolic compounds from agricultural waste can serve as natural preservatives in pharmaceutical formulations [69,70,71,72,73,74].

Table 4 offers various examples of specific types of agricultural residues, the bioactive compounds derived from them, and their extraction methods, based on pharmaceutical relevance.

Advanced techniques, such as microencapsulation and nanoencapsulation, have been demonstrated to notably enhance the bioavailability, solubility, and stability of bioactive compounds derived from agricultural residues and are decisive for their effective utilization in pharmaceutical applications [89,90,91].

The primary distinction between microencapsulation and nanoencapsulation lies in particle size, with microencapsulation typically ranging from 1 μm to 1 mm, and nanoencapsulation involving even smaller particles. Both techniques rely on particle dimension and distribution homogeneity for effectiveness. The supercritical carbon dioxide use in encapsulation processes offers meaningful advantages in designing particle size and managing drug-loading procedures under various conditions. Nanoencapsulation, in particular, prevents the degradation of active pharmaceutical ingredients and improves the accuracy of drug delivery by enabling proper cell entry through surface coating or conjugation. Additionally, nanoencapsulated pharmaceuticals can be labeled with fluorescent probes, assessing therapeutic efficacy during preclinical and clinical research [92,93,94,95]. These methods involve capturing the bioactive molecules within protective matrices, which defend them from degradation during processing and storage and, in addition, facilitate controlled release and targeted delivery in the human body. Moreover, novel carriers such as nanoparticles and liposomes can improve the absorption and bioactivity of the active substances, thereby maximizing their therapeutic potential. Additionally, these compounds’ incorporation into biodegradable polymers ensures sustained release, further increasing their efficacy [1,96].

Table 5 provides a few examples of encapsulation techniques for the phytochemicals retrieved from agro-residue.

In addition to the encapsulation approaches, emulsion-based systems successfully increase the efficiency and stability of compounds derived from agricultural residues for pharmaceutical applications. For instance, mango peel phenolics were effectively encapsulated in water-in-oil-in-water emulsions using various surfactants. Moreover, the use of oil-in-water excipient emulsions prepared by microfluidics has been shown to increase the total phenolic content and lycopene bioaccessibility in tomato pomace [103,104].

Biotechnological approaches, particularly fermentation, are extensively utilized to valorize agricultural residues by converting them into functional components. Fermentation is one of the oldest and most efficient methods for enhancing the content and bioavailability of bioactive compounds through anaerobic metabolism. This process is highly favored in both scientific and industrial fields due to its low energy consumption, minimal water generation, and cost-effectiveness. Three main types of fermentation—solid-state, submerged, and liquid—are employed based on the desired product. Among these, solid-state and submerged fermentation are most used in current research and industry for the extraction of bioactive compounds. Recent advancements in fermentation processes have focused on producing important bioactive compounds, such as antioxidants, which are increasingly recognized for their nutritional and health benefits, making this approach particularly relevant for pharmaceutical applications [105,106,107,108].

Table 6 displays certain examples of fermentation methods used on specific agricultural wastes.

Due to these modern technologies, the pharmaceutical industry can develop more efficient and stable formulations, therefore exploiting the full potential of the agricultural residues [10].

## 5. Pharmaceutical Applications of Upcycled Agricultural Residues

Pharmaceutical applications of upcycled agricultural residues represent a novel and challenging domain for researchers globally. Annually, billions of tons of agricultural wastes are produced, offering substantial potential for utilization across various industries. However, not all agricultural products are suitable for use due to their toxicity; for instance, β-hydroxy acids pose potential health risks to humans [113]. Despite these limitations, numerous agricultural residues from fruits and vegetables exhibit numerous pharmaceutical advantages in chronic diseases such as diabetes, cardiovascular diseases, and cancer management and treatment strategy [114,115]. Waste from the agriculture industry also can enhance the body’s absorption of various pharmaceuticals. These residues are an excellent source of essential nutrients and phytochemicals. The phenolic compounds are valuable for their therapeutic applications, including anti-inflammatory, antibacterial, antioxidant, and anticancer properties. Moreover, their antibacterial effect is enhanced by various antibiotics produced from microbes cultivated on agricultural waste [70,74].

Extensive research has been conducted on the bioactive compounds present in agricultural residues. For example, the industrial processing of oranges and lemons generates millions of tons of waste annually, rich in hydroxycinnamic acids and flavonoids [116]. Potato peels, a prevalent vegetable residue, contain chlorogenic acid as the most abundant phenolic acid and have numerous potential applications [117]. Lignocellulosic agricultural byproducts, such as wheat and rice bran, spent coffee grounds, and wheat straw residues, are recognized as clean sources of phenolic compounds, offering antioxidant and antimicrobial properties suitable for various functions [116].

**Figure 3:**
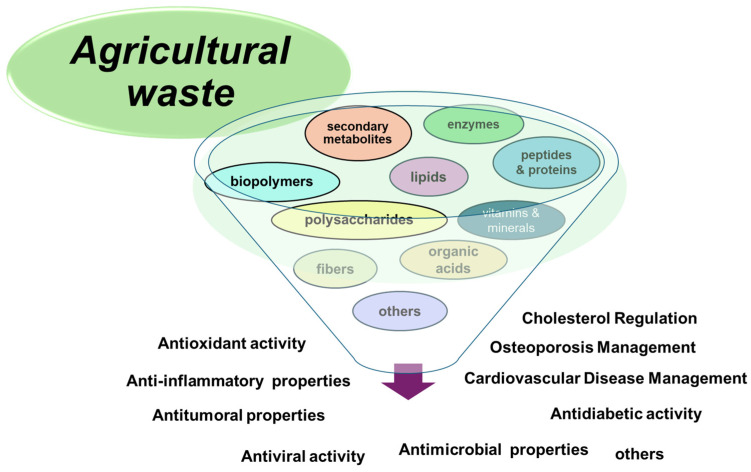
The main biomedical applications of agricultural waste.

Figure 3 shows several pharmaceutical applications of agricultural residues.

Table 7 offers some examples of agricultural byproducts, according to their relevance in the pharmaceutical field [113].

### 5.1. Antioxidants

Antioxidant activity is defined as the inhibitory effect of certain compounds in protecting the organism from free radicals. These radicals, which can originate within the body or be produced by external sources such as pollution, pose significant risks, including cancer and degenerative diseases such as ocular diseases, Alzheimer’s disease, and atherosclerosis [118,119,120]. The mechanism involves the radicals attacking macromolecules within the organism, leading to cellular damage, including damage to proteins and deoxyribonucleic acid (DNA) [121].

The human body can obtain natural antioxidants from fruits and vegetables as valuable sources of health-beneficial phytochemical compounds. Phytochemicals derived from agricultural residues possess antioxidant capacities and are beneficial in managing chronic diseases associated with oxidative stress [120,122]. Agricultural residues from fruits can be utilized to obtain phytochemical compounds with antioxidant properties. Phenolic acids are among the most potent antioxidant elements [113,119]. For instance, phenolic acids with high absorption in the digestive tract can be extracted from the peels of oranges, lemons, grapefruits, grapes, apples, and bananas [122]. From vegetables such as sugarcane, maize, potatoes, soybeans, and tomatoes, various phenolic acids can be extracted. These acids are present in the bagasse, pollen, husks, peels, seeds, leaves, and pulp [113]. For example, potatoes contain a significant quantity of phenolic acids, and it is projected that by 2030, potato peel waste will reach up to 8000 kilotons globally [123]. Furthermore, studies indicate that red beet is among the top vegetables as regards the antioxidant provision [124]. Benzoic acid, gallic acid, and vanillic acid are among the most common phenolic acids, playing a crucial role in neurodegenerative disease treatment associated with oxidative stress and the body’s immune response. Their mechanism of action involves breaking the chain reactions and capturing free radicals from reactive oxygen species [125,126].

A meaningful volume of residue discarded from olives consists of their leaves and seeds, rich in biomolecules such as oleuropein, hydroxytyrosol, and tyrosol [127]. However, a study determined that while olive leaves exhibit antioxidant properties, chestnut and plum leaves possess significantly greater antioxidant activity than olive leaves [128]. Additionally, vitamins, carotenoids, and flavonoids exhibit antioxidant properties, each employing different mechanisms to defend against free radicals. These compounds are found in various parts of fruits (such as peels, seeds, pulp, skin, leaves, flowers, and stems) and vegetables (including pomace, leaves, bagasse, cobs, fiber, and seeds) that are considered waste [129].

Duda-Chodak and Tarko examined the antioxidant capabilities of fruit seeds and peels, demonstrating that the peels exhibit the highest antioxidant activity and polyphenol concentration [130]. Pomegranate farming generates enormous quantities of residues containing punicalin, punicalagin, and ellagitannins, which exhibit potent antioxidants [131]. Overall, phenolic compounds are especially significant because of their well-documented beneficial results on human health, including their roles in cancer and cardiovascular disease avoidance. These actions are attributed to their ability to act as effective antioxidants, counteracting oxidative stress considered the principal cause of various inflammatory and degenerative conditions [132].

### 5.2. Antitumor Agents

The antitumor mechanism is closely linked to antioxidant activity, as phenolic acids originate from fruit and vegetable residues, combat free radicals, and protect the body against cancer, particularly colon adenocarcinoma (Table 8) [133,134].

### 5.3. Cardiovascular Drugs

Many fruit and vegetable residues include compounds with important activity against cardiovascular diseases. Apple pomace contains uric acid, which exhibits endothelial reactivity against xanthine oxidase, providing protective effects [144]. In addition to its antioxidant properties, resveratrol from grape seeds has demonstrated efficacy in managing cardiovascular diseases by reducing atherosclerosis, acting on the renin–angiotensin system, and enhancing nitric oxide production. Procyanidins extracted from grape pomace have been shown to reduce aortic atherosclerosis [141,142]. Olive leaves consist of compounds such as uvaol, ursolic acid, and oleanolic acid, which have vasodepressor effects and help control hypertension by lowering systolic and diastolic blood pressure [127]. Pineapple peels contain bromelain, which has antithrombotic properties and inhibits platelet aggregation [145]. Flavonoids from various fruit and vegetable residues are also effective in managing atherosclerosis by preventing the oxidation of LDL lipoproteins [146]. Polyphenols exhibit antithrombotic activity, and quercetin from onion residues has been shown to act against atheroma plaque formation [147].

### 5.4. Antidiabetic Compounds

Several compounds extracted from agricultural residues exhibit significant antidiabetic effects. Chlorogenic acid and quercetin, extracted from apple skins, regulate glucose absorption, thereby reducing postprandial glycemia. Moreover, phytochemicals from apple pomace have been shown to influence hyperglycemia and insulin resistance [144]. Oleuropein, derived from olive leaves, can decrease blood glucose levels [127]. Limonin, found in citrus peels, exhibits anti-diabetic activity by affecting relevant biomarkers [143]. Kaempferol, a flavonol compound, has recently been discovered to reduce hyperglycemia [128]. Bell pepper residues, including seeds, peels, and leaves, contain antioxidant compounds that protect against oxidative damage and enhance the sensitivity of pancreatic β-cells to glucose [148]. Furthermore, catechin, isoflavones, tannic acid, and saponins from various fruit and vegetable residues contribute to glucose transport regulation [147].

### 5.5. Cholesterol Regulation

Apple pomace contains phloridzin and catechin, which have been shown to decrease triglycerides and LDL-cholesterol levels [144]. Resveratrol from grape seeds also exhibits hypolipidemic activity [141,142]. Compounds found in avocado leaves can reduce LDL cholesterol and total cholesterol [136]. Pomegranate seeds contain punicic acid, which helps decrease hyperlipidemia. Additionally, allyl methyl sulphonate and γ-glutamyl cysteine from raw garlic homogenate inhibit LDL oxidation, reducing fatty streak deposition in blood vessels [149].

### 5.6. Anti-Inflammatory Agents

The phenolic compounds, flavonoids, and tannins extracted from agricultural wastes exhibit strong anti-inflammatory properties in the human body [146]. Furthermore, polyphenols from fruits and vegetables possess both anti-inflammatory and antioxidant properties, which contribute to their effectiveness as anti-aging agents [147]. Olive leaves consist of quercetin, which has properties beneficial for the treatment of gastric ulcers [127].

### 5.7. Osteoporosis Management

Citrus peels, such as those from limes and oranges, comprise hesperidin, which has properties that can help decrease the risk of osteoporosis or alleviate its symptoms [145].

### 5.8. Antimicrobial/Antiviral Drugs

Various agricultural residues from fruits and vegetables exhibit antimicrobial activity against pathogens, including fungi and viruses. For instance, apple pomace contains phenolic compounds that possess antioxidant properties and antiviral activity against the *Herpes simplex* virus [150]. Research highlights the importance of flavonoids, phenolic acids, and other compounds in apple pomace for combating microbial and viral pathogens such as *Paenibacillus larvae*, *Escherichia coli*, *Pseudomonas aeruginosa*, *Streptococcus pyogenes*, *Enterococcus faecalis*, and *Staphylococcus aureus* [144]. Orange peel has demonstrated activity against *S. aureus* and *Candida albicans* [128]. Olive leaves exhibit antimicrobial properties due to compounds like verbascoside, oleuropein, and luteolin, showing higher activity against *Bacillus cereus*, *C. albicans*, and *E. coli*, and lower activity against *Klebsiella pneumoniae* and *P. aeruginosa* [151].

Flavonoids and anthocyanins from agricultural residues are recognized as therapeutic compounds with antifungal and antibacterial properties. Tangeretin and hesperidin from citrus peels show antiviral properties against hepatitis B virus by blocking viral fusion in the host organism [145]. Hesperidin also exhibits activity against the severe acute respiratory syndrome coronavirus 2 (SARS-CoV-2) by binding to and inhibiting the spread of viral cells [152]. Banana peel contains tannins with antimicrobial properties and other compounds active against *S. aureus*, *Salmonella enteritidis*, and *E. coli*, and also shows biofungicidal activity [153].

Phenolic compounds found in many agricultural residues have bactericidal, antiseptic, and anthelmintic properties. Additionally, saponins from these residues exhibit antimicrobial properties [146].

Various agricultural byproducts are utilized in the synthesis of antibiotics. Researchers found that peanut shells were the most productive substrate for generating tetracycline, followed by corncobs [154].

Utilizing low-cost carbon sources extracted from diverse agricultural residues made antibiotic manufacturing far more affordable, offering remarkable chances for innovation in antibiotics such as neomycin. Additionally, the synthesis of extracellular rifamycin B using solid-state fermentation with oil-pressed cake, an agro-industrial waste, was considered [155]. Another study focused on producing oxytetracycline from cocoyam peels, through solid-state fermentation by *Streptomyces speibonae* [156].

### 5.9. Skincare Formulations

Certain fruits and vegetables hold significant importance in the beauty and pharmaceutical industries. For instance, compounds with moisturizing and antioxidant properties can be extracted from rice, orange, and oat bran, which are beneficial for skincare [145].

Skin tone is particularly important in contemporary society, especially for women, who often perceive variations in skin color (ranging from low to high yellow or red tones) as indicators of health and attractiveness. Carotenoids extracted from carrots, tomatoes, and other red vegetables can enhance skin color and improve its appearance [157].

Resveratrol and vitamin C extracted from grape seeds possess the ability to reduce melanin concentration in the skin, making them effective skin-lightening agents. In the pharmaceutical domain, these compounds are particularly important for addressing hyperpigmentation caused by UV radiation or hormonal changes [158].

From cocoa pods, malic acid, rosmarinic acid, and ellagic acid can be extracted. These acids exhibit high antioxidant activity and are included in anti-wrinkle products in the pharmaceutical industry due to their ability to enhance skin hydration [159,160]. Additionally, polyphenolic extracts from apples possess anti-aging properties and can regulate sebum production in acne, thereby reducing dermal inflammation [161].

## 6. Limitations

Despite the promising prospects, several limitations delay the upcycling of agricultural residues for pharmaceutical applications. One major challenge is the variability in the composition of agricultural residues, which affects the consistency and quality of extracted compounds. Factors such as geographic location, seasonal changes, and agricultural practices contribute to this inconsistency. In addition, scaling up biotechnological processes from laboratory to industrial proportion presents major technical and economic challenges. The high costs associated with advanced extraction technologies and the need for rigorous quality control measures further complicate the commercialization of these bioproducts [162,163].

Another critical limitation is the need for strong regulatory strategies to support the use of agricultural residues in pharmaceutical applications. Ensuring the safety, efficacy, and quality of bioactive compounds necessitates complex guidelines, accurate testing, and standardized protocols. These frameworks are essential to guarantee that the phytochemicals derived from agricultural residues meet the high standards required for pharmaceutical use, eliminating toxic substances and preventing microbial hazards [164].

Environmental concerns are also relevant: while upcycling reduces waste, the systems involved must be managed to minimize environmental impact. Developing green technologies and sustainable practices will be essential for the long-term viability of these initiatives (Table 9).

## 7. Conclusions and Future Perspectives

The upcycling of agricultural residues into beneficial pharmaceutical products holds great promise for future developments. Biotechnological methods, such as fermentation and enzymatic hydrolysis, are expected to play a vital role in extracting valuable bioproducts from agricultural waste. The integration of nano- and biotechnological methods is particularly promising due to their low energy requirements and cost-effectiveness, making them ideal for industrial residue valorization [10].

Advances in these domains could lead to the development of unique biocompounds with applications across various industries, including pharmaceuticals. Furthermore, future research should focus on optimizing eco-friendly and cost-effective conversion techniques, exploring underutilized agricultural residues, and identifying novel bioactive molecules with potential health benefits [91,165].

Emphasis should also be placed on creating innovative encapsulation procedures to improve the stability and bioavailability of these compounds, thereby maximizing their therapeutic efficacy. Moreover, supporting collaborations between scientists, the pharmaceutical industry, and government agencies will be important in accelerating the laboratory-scale innovations translation to commercial-scale applications.

Agricultural residue upcycling presents a promising avenue for sustainable waste management and valuable pharmaceutical product production. This review highlights the momentous potential of agricultural byproducts as sources of bioactive compounds, which can be utilized in various therapeutic applications, including antioxidants, anticancer agents, and anti-inflammatory drugs, as well as in antibiotic manufacturing. Progress in biotechnological and nanotechnological methods has facilitated the efficient extraction and enhancement of these compounds, making their industrial application more feasible and cost-effective. Despite the challenges associated with the variability in residue composition, scalability, and regulatory frameworks, the continued development of eco-friendly extraction techniques and robust administrative standards will be crucial. Future research should focus on optimizing these processes, exploring underutilized residues, and encouraging interdisciplinary collaborations to fully access the potential of agricultural waste. By addressing these challenges, the pharmaceutical industry can fundamentally benefit from the sustainable and innovative use of agricultural residues, contributing to both environmental conservation and public health advancements.

## Figures and Tables

**Table 1:** Primary types of agro-waste.

Livestock Waste	Crop Waste	Hazardous and Toxic Agricultural Waste
manure, animal carcasses	drops and culls from fruits and vegetables	herbicides
urine, dung, wash water, residual milk, waste feed	leaf litter, seed pods, stems, straws, husks, weeds	pesticides
poultry waste (spilled feed, feathers, droppings, bedding material)	agro-industrial waste (bagasse, molasses, peels, pulps, oil-seed cakes)	insecticides
slaughterhouse waste (blood, hair, hides, flesh, bones)	sugar cane bagasse	
aquaculture wastes (uneaten feed, fecal waste)	corn stalks, pruning	

**Table 2:** Agricultural and food processing residues.

Food Processing Waste	Major Nutritional Groups Contributing to Waste	Specific Food Industry Wastes	Vegetable, Fruit, and Crop Processing Wastes
residues in solid, liquid, or mixed state during processing	oil-bearing crops, cereals, tubers, pulses, roots, vegetables, and fruits; meat and animal products	shells, heads, scales, tails of marine animals; offal, fats, internal organs, blood, lard of animals; milk sediment, curd, cheese whey	pulp, bran, pomace, peels, cull, seeds, stems, germs, shells

**Table 3:** Valorization techniques for transforming agricultural residues into pharmaceutical bioproducts.

Valorization Technique	Recovery of Biocompounds for Pharmaceutical Applications	Technical Details (Advantages/Limitations)	Ref.
enzymatic hydrolysis (biological)	✓ antibiotics production	➢ efficient under mild conditions with minimal effort ➢ low hydrolysis rate; ➢ requires a large sterile space	[26,27,28,29]
green solvents (natural deep eutectic solvents, ionic liquids)	✓ employed in the production of pharmaceuticals with antioxidant, antibacterial, and anticancer properties	ionic liquids ➢ effective at dissolving cellulose; ➢ limited by toxicity, high costs, ➢ impractical for large-scale; deep eutectic solvents ➢ environmentally friendly, safe ➢ can produce contaminants, ➢ higher viscosity; natural deep eutectic solvents ➢ low-cost, available, modifiable, ➢ less hazardous, ➢ favorable for industrial use	[30,31,32]
microwave processing, including microwave pyrolysis (physical)	✓ use to enhance the breakdown of agricultural waste into simpler compounds that can be further used in pharmaceutical applications; ✓ efficiently operational, capable of processing large quantities of residue with minimal formation of inhibitors	➢ efficient and handles large agro-waste with few inhibitors, ➢ require increases temperature and electricity.	[33,34,35,36]
pyrolysis (physical)	✓ useful in transforming agricultural residue into bio-oil and biochar which can be consequently processed into pharmaceutical-grade bioactive compounds, such as nutraceuticals, ethanol, and enzymes; the maximum achievable rate of conversion from cellulose to sugars	➢ achieves the highest cellulose sugar conversion rates, ➢ is a high-cost technique	[37]
ultrasonic processing (physical)	✓ used to increase the decomposition efficiency of lignocellulosic materials, thereby aiding in the extraction of essential pharmaceutical substances	➢ facilitates lignocellulosic breakdown, ➢ can cause antagonistic effects from prolonged sonication	[38]
acid hydrolysis (chemical) (hydrochloric acid, acetic acid, and sulfuric acid)	✓ enables the conversion of intricate lignocellulosic structures into basic sugars essential for pharmaceutical production (including the generation of carbohydrates such as glucose, mannose, xylose, and galactose, along with organic acids like acetic acid and formic acid, critical for the synthesis of bioactive compounds)	✓ changes lignin structure and hydrolyzes hemicellulose effectively ✓ causes equipment corrosion and produces harmful byproducts	[39]
alkaline hydrolysis (chemical) (potassium hydroxide, sodium hydroxide, ammonium hydroxide, magnesium hydroxide, and calcium hydroxide)	✓ enhances the digestibility of agricultural residue, making it suitable for additional processing into bioactive compounds for medicinal use; ✓ pretreatment under gentler conditions, facilitates the removal of lignin and hemicelluloses, thereby increasing the available surface area	✓ removes lignin and hemicellulose under mild conditions, increasing surface area ✓ requires high alkalinity and long processing times	[21,40]
ozonolysis (chemical)	✓ employs ozone to degrade lignin, making the agro-waste components more amenable to pharmaceutical exploitation	✓ reduces lignin without hazardous byproducts ✓ is expensive and demands large amounts of ozone	[41]
organosolv (chemical)	✓ involves the use of organic solvents to extract valuable pharmaceutical precursors, such as phenolic compounds and acids from agro-waste materials	➢ hydrolyzes lignin and hemicellulose effectively, ➢ costly solvents limit industrial use due to high volatility	[42]
wet oxidation (chemical)	➢ applied to effectively remove lignin, thus enriching the agricultural residue with compounds that can be transformed into drugs	➢ effectively removes lignin with low inhibitor formation, ➢ is costly due to oxygen and acid catalyst use	[43]

**Table 4:** Bioactive compounds from agricultural residues.

Type of Residue	Bioactive Compounds	Extraction Methods	Technical Details	Ref.
tomato processing residue	lycopene, β-carotene	➢ ultrasound-assisted extraction; ➢ conventional organic solvent extraction	✓ solvent mixture of hexane–acetone–ethanol (2:1:1, *v*/*v*/*v*) with 0.05% (*w*/*v*) butylated hydroxytoluene (BHT), solid–liquid ratio of 1:35 (*w*/*v*), at 15 °C, 90 W for 15 or 30 min	[75]
orange peel	pectin, flavonoids, hesperidin, polyphenols	➢ microwave-assisted extraction; ➢ hot-water extraction ➢ rapid solid-liquid dynamic (RSLD) extraction	✓ acidic hot-water extraction: liquid-to-solid ratio (20), pH 1.5 at 70 °C for 60 min	[76]
grape pomace	anthocyanins, polyphenols	➢ ultrasound-assisted extraction; ➢ microwave-assisted extraction	✓ microwave-assisted extraction with 2% citric acid at 1000 W for 10 min	[77]
banana peel	pectin, polyphenols	➢ extraction with hydrochloric acid or citric acid	✓ 0.5 N HCl at 90 °C for four hours	[78]
kiwi juice pomace	polyphenols, flavan-3-ols, ascorbic acid	➢ microwave-assisted extraction	✓ microwave power of 400 W; pressure of 350 psi; solvent composition: 50% ethanol–water, solid-to-solvent ratio: 1:15 at 75 °C for 15 min	[79]
rice bran	oils (tocopherols, tocotrienols, oryzanols)	➢ conventional Soxhlet extraction; ➢ subcritical CO_2_ Soxhlet extraction	✓ subcritical CO_2_ Soxhlet extraction: solvent-to-feed ratio 24:1 at 68–70 bar and 27–29 °C	[80]
pistachio hard shells	polyphenols, flavonoids (gallic acid, monogalloylglucose isomer, pentagalloylglucose, kaempferol)	➢ microwave-assisted extraction; ➢ extraction with various solvents	✓ microwave-assisted extraction using ethanol at 1000 W for 270 s	[81]
pumpkin seeds	oils (palmitic acid, oleic acid, linoleic acid, saturated fatty acids, monounsaturated fatty acids, and polyunsaturated fatty acids), proteins, polysaccharides	➢ ultrasound-assisted three-phase partitioning extraction	✓ (NH_4_)_2_SO_4_ addition 30 g/100 mL, and *t*-butanol to slurry ratio of 1.0:1.0 (*v*/*v*), pH 5, and a duty cycle of 60% at 118 W for 20 min irradiation time	[82]
hot pepper paste pulp	capsaicin, polyphenols	➢ ultrasound-assisted extraction; ➢ maceration extraction	✓ ultrasound-assisted extraction: 60% amplitude, 60 °C for five minutes; maceration extraction for eight hours, 50 °C	[83,84]
coffee husk	caffeine	➢ supercritical CO_2_ extraction	✓ solvent to raw material mass ratio: 197 kg CO_2_/kg husks at 373 K and 300 bar	[85]
red apple peel	anthocyanins	➢ extraction	✓ reflux, 2 M HCl	[86]
beetroot residue	polyphenols (ferulic acid, vitexin, sinapaldehyde)	➢ pressurized liquid extraction	✓ 40 °C for 90 min with mixture of ethanol–water (70:30, *v/v*) at 10 MPa	[87]
sesame bran	proteins, antioxidants	➢ enzyme-assisted extraction; ➢ ultrasound-assisted extraction	✓ pH 9.8; 1.248 Anson Unit (AU)/100 g enzyme concentration at 836 W, 43 °C for 98 min	[88]

**Table 5:** Extracted bioactive compounds and their encapsulation method.

Encapsulation Technique	Phytoconstituents Extracted	Technical Details	Ref.
extrusion	phenolic compounds from winemaking residue	➢ efficient wall material: mixture of 1% (*w*/*v*) alginate and 3% (*w*/*v*) chitosan, encapsulating about 80% of extracts, ➢ retains chemical stability and biological activities	[97]
spray/freeze drying	carotenoids from carrot processing residue	➢ freeze-drying: pure whey protein (63.69 g/100 g) produces encapsulate with best hygroscopicity, oxidative stability, antioxidant capacity, and color properties; spray drying: mixture of 71 g/100 g whey protein and 29 g/100 g inulin, ➢ produces encapsulate with lowest water activity, moisture content, and particle size	[98]
freeze drying	bioactive compounds from beetroot pomace	➢ freeze-dried encapsulate: lower moisture content and water activity than spray-dried, retains 76.67% polyphenols, betalain pigments, 17.77% betacyanins, and 17.72% betaxanthins during three months storage at room temperature; ➢ higher release of polyphenolic compounds in simulated intestinal fluid than in gastric fluid during in vitro digestion	[99]
electrospinning	carotenoids from tomato peels	➢ nanoencapsulation process: increases 11 times the antioxidant activity; ➢ better retention of lycopene and antioxidant activity during 14-day storage compared to nonencapsulated extract	[100]
spray/freeze drying	carotenoids and phenolic compounds from sweet potato peels	➢ freeze-drying yields better water activity, moisture content, hygroscopicity, and encapsulation efficiency of phenolics; ➢ spray drying produces smaller particle size, better flow properties, and encapsulation efficiency of carotenoids	[101]
spray/freeze/microwave drying	phenolic compounds from cornsilk	➢ freeze-drying with 100% maltodextrin: ➢ achieves highest recovery of phenolic compounds and retention of antioxidant activity	[102]

**Table 6:** Fermentation of agricultural residues.

Fermentation Method	Agro-Residue	Technical Details	Ref.
solid-state fermentation with *Rizhopus oryzae*	rice bran	➢ doubles the phenolic compound content, with highest increase in gallic and ferulic acid; ➢ phenolic extract from fermented rice bran inhibits peroxidase enzyme	[109]
solid-state fermentation with *Aspergillus niger* and *R. oligosporus*	apricot pomace	➢ total phenolic and flavonoid contents increased significantly: by more than 70% for total phenolics and 38% for total flavonoids using *R. oligosporus*, and by more than 30% for total phenolics and 12% for total flavonoids using *A. niger*	[110]
fermentation with *Lactobacillus plantarum*	tomato seeds	➢ reduction in crude and soluble protein contents: 18.44% and 68.99%, respectively, after 24 h of *L. plantarum* growth on the substrate	[111]
solid-state fermentation with *A. niger*	mango seeds	➢ enhancement of polyphenolic compound mobilization; ➢ improvement of nutraceutical characteristics	[112]

**Table 7:** Agricultural residues and their primary pharmaceutical applications.

Agricultural Residue	Pharmaceutical Applications of Recovered Biomolecules
apple pomace	➢ contains phloridzin and catechin; ➢ used to decrease triglycerides and low-density lipoprotein (LDL)-cholesterol levels and to manage diabetes.
grape seeds	➢ rich in resveratrol; ➢ effective for cardiovascular disease management and exhibits hypolipidemic activity
olive leaves	➢ contains oleuropein, uvaol, ursolic acid, and oleanolic acid; ➢ used for reducing blood glucose levels and controlling hypertension
citrus peels	➢ source of hesperidin; ➢ has anti-osteoporosis properties and flavonoids for prostate and colon cancer management
pomegranate byproducts	➢ contains ellagitannins, punicalagin, and punicalin; ➢ exhibits antioxidant and anti-cancer activities
potato peels	➢ rich in chlorogenic acid and glycoalkaloids; ➢ used for anti-inflammatory, antibacterial, and hepatoprotective effects
papaya seeds	➢ contains isothiocyanate; ➢ inhibits tumor formation and exhibits activity against lung, colon, breast, and prostate cancer
avocado residues	➢ seeds contain flavonoids and tannins with anticancer properties; ➢ leaves contain persin, which induces apoptosis in breast cancer cells
rice husk	➢ contains peptides that activate anticancer mechanisms in the body
banana peels	➢ rich in tannins and other compounds; ➢ exhibits antimicrobial properties and antioxidant activity

**Table 8:** Examples of agricultural residues with anticancer activity.

Types of Agriculture Residues	Recovered Phytochemicals	Antitumor Activity	Ref.
pomegranate fruit residues (peels and seeds)	various bioactive compounds	lung cancer	[135]
avocado seeds	flavonoids and tannins	prevent tumor growth	[128]
avocado leaves	persin	induce apoptosis in breast cancer cells	[136]
papaya seeds	isothiocyanates	inhibit tumor formation and display activity against lung, colon, breast, and prostate cancers	[137]
olive leaves	erythrodiol, uvaol, oleanolic acid, and maslinic acid	cytotoxic capacity against breast, colon, and cervical cancers	[127,128]
rice husks	peptides	can activate the body’s anticancer mechanisms	[138]
*Citrullus colocynthis* (stems, leaves, and roots)	alkaloids	liver, larynx, breast, pancreatic, and skin cancers	[139,140]
grape pomace (skin and seeds)	resveratrol	efficient against the proliferation and invasion of tumor cells	[141,142]
*Citrus* peels and seeds	polyphenolic compounds	prostate, colon, breast cancer	[114,143]
*Citrus* peels	limonin	human T-cell leukemia	[114,143]

**Table 9:** Knowledge gaps and recommendations for future studies.

Knowledge Gaps	Recommendations for Future Studies
variability in residue composition	➢ develop standardized methods for characterizing and processing residues to ensure consistent quality and efficacy of derived products
cost-effective extraction techniques	➢ research low-cost, efficient extraction methods that can be applied at an industrial scale
environmental impact	➢ explore the development of green technologies and sustainable practices to ensure the environmental benefits of upcycling outweigh the costs
regulatory frameworks	➢ establish clear guidelines and standardized protocols for the use of agricultural residues in pharmaceutical applications

## Data Availability

Data described in the manuscript will be made publicly and freely available without restriction at: https://docs.google.com/document/d/1yIATe2CRm1fEokLH8Qr7ZfasOw50KQKQ/edit?usp=sharing&ouid=106104952021876684289&rtpof=true&sd=true (accessed on 8 June 2024).
